# Differential Expression of Placental Glucocorticoid Receptors and Growth Arrest-Specific Transcript 5 in Term and Preterm Pregnancies: Evidence for Involvement of Maternal Stress

**DOI:** 10.1155/2014/239278

**Published:** 2014-05-11

**Authors:** D. Mparmpakas, E. Zachariades, G. Sotiriadis, A. Goumenou, A. J. Harvey, Y. Gidron, E. Karteris

**Affiliations:** ^1^Biosciences, Centre for Cell and Chromosome Biology, Brunel University, Uxbridge, UB8 3PH, UK; ^2^Department of Obstetrics and Gynecology, University Hospital, University of Crete Medical School, Heraklion, 71003 Crete, Greece; ^3^Biosciences, Brunel Institute for Cancer Genetics and Pharmacogenomics, Brunel University, Uxbridge, UB8 3PH, UK; ^4^Free University of Brussels (VUB), 1090 Jette, Belgium

## Abstract

Pregnancy-specific stress predicts birth outcomes. We hypothesized that there is a maternal stress-GR interaction that can influence fetal birth weight. This study examined the relationship between mothers' stress and attitude towards their pregnancies, placental glucocorticoid receptors (GRs) and growth arrest-specific transcript 5 (GAS5) expression, and the status of GR polymorphism, with their infants' birth weights. GAS5 and GR**α** were the predominant transcripts in both term and preterm placentas, with GAS5 being primarily localized in the syncytiotrophoblasts. In an attempt to mimic moderate and high stress environment *in vitro*, BeWo and JEG-3 cytotrophoblast cell lines were treated with 10 nM–1000 nM cortisol. Only expression of GAS5 was significantly upregulated by cortisol in all treatments compared with basal levels, but none of the GRs changed expression significantly. In an attempt to assess a stress versus gene interaction, we studied four GR polymorphisms. In the homozygous group for *Tth111*I polymorphism, mothers with negative attitudes towards the pregnancy gave birth to infants with significantly lower birth weights compared to women with positive/neutral attitudes. None of the GR splice variants were associated with maternal stress. However, placental GAS5 levels were inversely correlated with maternal stress. This study points towards a potential gene-environment interaction that could be of predictive value for fetal weight.

## 1. Introduction

Pregnancy is associated with major physiological and future psychosocial changes and adaptation to these changes is crucial for normal fetal development and for healthy infant-mother relationships. Maternal pregnancy-specific stress may be a more powerful contributor to birth outcomes than general stress [1]. Biologically, the stress hormone cortisol acts by activating glucocorticoid receptors (GRs). To date, four splice variants of the GR gene have been reported, formed by alternative splicing, termed GR*α*, GR*β*, GR*γ*, and GR-P [2]. Numerous studies have also reported the expression of different GR splice variants in different cell and tissue types [3–6]. Growth arrest-specific transcript 5 (GAS5) encodes a single strand noncoding RNA (ncRNA) and, as its name suggests, can accumulate in growth-arrested cells [7]. In a recent study by Kino and colleagues it was demonstrated that GAS5 ncRNA may be a repressor for the GR by acting as a decoy “glucocorticoid response element (GRE),” thus, competing with DNA GREs for binding to the GR. As a result, GRs cannot bind to their DNA GRE and subsequently their function is compromised [8].

Changes in the epigenetic regulation of the fetal GR promoter have been associated with exposure to prenatal maternal stress [9] reflecting a possible effect of maternal stress on the expression and function of the GR in the fetus. A GR* Bcl*I polymorphism has been associated with increased glucocorticoid sensitivity and was overrepresented in pregnant women with pathological, fetomaternal immune adaptation. Indeed, a number of polymorphisms have been described in the gene coding for the GR although it is still unclear whether the variability in the glucocorticoid responses observed is due to the polymorphisms or to other factors [10]. This sensitivity could be one mechanism linking maternal stress to fetal development. Only few of these polymorphisms are functionally relevant and these are the* Tth111*I, the ER22/23EK, the N363S, the—already mentioned—*Bcl*I, and the GR-9*β*. Studies have shown that at least three polymorphisms are associated with altered glucocorticoid sensitivity and also with changes in body composition and metabolic parameters [10, 11], which could affect fetal development as well.

This study examined the relationship between mothers' stress levels and attitude towards their pregnancies, placental GR and GAS5 expression, and the status of GR polymorphism, with their infants' birth weights. We also examined the maternal attitude versus gene interaction in relation to fetal birth weight. Moreover, we tested the effect of cortisol on GR splicing and GAS5 expression of BeWo and JEG-3 placental cells, in an attempt to resemble a low, moderate, and high stress milieu* in vitro*.

## 2. Methodology

### 2.1. Subjects

The study population consisted of pregnant women attending the Department of Obstetrics and Gynaecology, University Hospital, University of Crete. The participants were in the third trimester of their pregnancy. All participants gave informed consent to participate in the study and ethical approval was granted by the local ethics committee of the hospital. Questionnaires regarding maternal stress/maternal attitudes as well as anthropometric data were collected as previously described [12, 13]. Briefly, maternal stress/maternal attitudes towards their pregnancy were assessed in the 3rd trimester, using a questionnaire approach. A research nurse or a gynecologist conducted a face-to-face interview with each woman lasting approximately 20–30 minutes. Maternal attitudes were related to self-reported stress status ranging from low and medium (low stress response) to high and very high (high stress response). The exact cause of maternal stress was not identified. Maternal attitudes were divided into two groups: negative attitudes (48%) and positive (35.7%)/neutral attitudes (16.3%). Immediately after delivery the weight of the newborn was recorded.

### 2.2. Placental Tissue

Placental tissues were obtained from women delivering at term (>37 weeks of gestation) and preterm (<37 weeks of gestation) (total *n* = 23). Of the 13 term placentas 12 were in labor (mode of delivery: 10 caesarean sections [CS], 2 vaginal deliveries) and 1 nonlabor/CS tissue. None of the term or preterm women were administered any antidepressants or synthetic glucocorticoids during pregnancy. Of the 10 term placentas 4 were nonlabor and all CS, and 6 were from laboring tissues (mode of delivery: 3 CS, 3 vaginal deliveries). The average age was 29.6 ± 4 years, the mean pregnancy days were 240 ± 42, and mean fetal weight was 2805 ± 95 gr. After delivery, the maternal and fetal surfaces of the placenta were dissected, and fetal membranes were peeled away from the placenta. Placental samples were washed in PBS and immediately stored in RNAlater (Applied Biosystems, UK) at −80°C. Ethical approval was granted from the local ethics authority.

### 2.3. Cell Culture

BeWo and JEG-3 cell lines were maintained at standard culture conditions of 5% CO_2_ in air at 37°C. BeWo cells were cultured in Ham F12 (Sigma-Aldrich, UK) containing 10% heat-inactivated fetal bovine serum (FBS) and 0.5% penicillin streptomycin, whereas JEG-3 cells were maintained in MEME (Sigma-Aldrich, UK) containing 10% heat-inactivated FBS and 0.5% penicillin streptomycin, 0.5% L-glutamine, 0.5% sodium pyruvate, and 0.5% MEM nonessential amino acids. Prior to cortisol treatment, both cell lines were maintained for 3 hours in phenol red-free media containing charcoal stripped FBS.

### 2.4. RNA Isolation, cDNA Synthesis, and PCR

Total ribonucleic acid was isolated using an RNA extraction kit (Sigma-Aldrich, UK), according to manufacturer's instructions. RNA concentration was determined by spectrophotometric analysis (NanoDrop; Thermo Scientific, UK) and agarose gel electrophoresis. RNA (200 ng from placental tissue and 500 ng from cell lysates) was reverse-transcribed into cDNA using 5 IU/*μ*L RNase H reverse transcriptase (Invitrogen, Paisley, UK).

### 2.5. Quantitative RT-PCR

Relative expression of the genes of interest was assessed by quantitative PCR (Q-PCR) on an ABI Prism 7900HT Sequence detection system (Applied Biosystems) using SYBR Green-PCR reaction mixture (Sigma-Aldrich, UK) and specific primers (Table 1) as previously described [14]. For the quantitative PCR, the following equations were used: ΔCt = Ct_(gene  of  interest)_ − Ct_(house  keeping  gene)_, ΔΔCt = ΔCt_(sample)_ − ΔCt_(calibrator)_, and Relative Quantity (RQ) = 2^−ΔΔCt^ as previously described [12].

### 2.6. Immunofluorescent Analysis

Following a series of deparaffinisation and dehydration, placental tissue sections were incubated with 10% bovine serum albumin (BSA) for 1 hr. This was followed by incubation for 1 hr with antibodies against GR*α* at a 1 : 200 dilution in 1% BSA/PBS. Cells were then washed with PBS prior to an incubation with a TRITC-conjugated secondary antibody (Santa Cruz Biotechnology, USA) for 1 hr. Slides were washed with PBS and mounted in Vectashield Mounting Medium (Vector Labs) containing the dye 4,6-diamido-2-phenylindole (DAPI) to counterstain nuclei. Images were captured using a Plan Apo Neofluor 63X NA 1.25 oil objective (Zeiss) on a Zeiss Axiovert 200M microscope and viewed using the AxioVision software.

### 2.7. Western Immunoblotting

Proteins from placental lysates and BeWo cells treated with 10–1000 nM cortisol were separated on an SDS-12% polyacrylamide gel and transferred to a nitrocellulose membrane. The membrane was blocked in TBS containing 5% dried milk powder (w/v) and 0.1% Tween-20, overnight at 4°C. After three washes with TBS-0.1% Tween-20, the nitrocellulose membranes were incubated with primary antibodies against GR*α*/*β* (Abcam, ab3580, UK) and GAPDH (Sigma-Aldrich, UK). The primary antisera were used at a 1 : 1000 dilution for GR*α*/*β* and 1 : 2000 for GAPDH, overnight at 4°C. The membranes were washed thoroughly for 30 min with TBS-0.1% Tween, before incubation with the secondary HRP-conjugated immunoglobulin (1 : 2000) for 1 hr at room temperature and further washing for 30 min with TBS-0.1% Tween-20. The immunoreactive bands were analyzed using Image J 1.34s image-analysis software (National Institute of Health, USA).

### 2.8. RNA Fluorescent In Situ Hybridization (FISH)

The slides of the paraffin embedded placental tissue samples were placed in a coplin jar and deparaffinised for 30 minutes at 37°C using Histo-Clear (Fisher Scientific, UK). Thereafter, the tissue sections were rehydrated by placing the slides in ethanol solution of different concentrations (100%, 90%, 80%, 70%, 50%, and 30%) for 3 minutes. A brief wash in 1x PBS solution was performed after this. Pepsin (0.01% in 0.01 M HCl) was used to treat the tissue for 5 minutes at 37°C, and a brief rinse in DEPC treated H_2_O followed. Then, the tissue samples were dehydrated by placing them in ethanol solutions (70%, 90%, and 100%) for 5 minutes. A specific Alexa 488-conjugated GAS5 hybridization probe (GTGCTATCCAGAGCCACACTGCATCTGCACCCAGCACCATACCTCACAG) was utilised as previously described [8] and followed by an overnight incubation at 37°C in a humidified chamber. After this incubation, three 10-minute washes were followed in 2xSSC at 37°C. The slides were then briefly rinsed in DEPC treated H_2_O and mounted in Vectashield Mounting Medium containing DAPI prior to examining the emitted fluorescent signal under a Zeiss Axiovert 200M microscope viewed using the AxioVision software.

### 2.9. GR Polymorphisms

For the* Bcl*I, N363S,* Tth111*I, and ER22/23EK GR polymorphisms DNA was extracted using the Phusion Blood Direct PCR Kit (New England Biolabs, UK), according to the manufacturer's instructions. For the* Bcl*I, the following primers were used: 5′-AAGCTTAACAATGGCCAT-3′ and 5′-TGCTGCCTTATTTGTAAATTCGT-3′. The PCR conditions used were 40 cycles of 98°C for 5 sec, annealing at 50°C for 5 sec, and elongation at 72°C for 15 sec. To confirm the presence of the* Bcl*I polymorphism, 17 *μ*L of the PCR product was digested with 2 *μ*L of 10x Restriction Endonuclease Buffer and 15 U of the restriction enzyme* Bcl*I (New England Biolabs, UK) for 1.5 hr at 50°C. The resulting digested fragments were separated on a 2% agarose gel to determine the genotypes. For the N363S polymorphism, specific PCR primers were used: 5′-AGTACCTCTGGAGGACA G AT-3′ and 5′-GTCCATTCTTAAGAAACAGG-3′ under the same PCR conditions. The restriction enzyme Tsp509I (10 U) was used to digest the PCR products at 65°C for 4 hours. For the ER22/EK23 polymorphism, we have used the following primers: 5′-GATTCGGAGTTAACTAAAAG-3′ and 5′-CTACCCTTTACTGGACCCTA-3′, followed by restriction digest using MnI I (New England Biolabs, UK) at 37°C for 4 hours. Finally, the* Tth111*I polymorphism was assessed using the PCR primers: 5′-GGCCACAACAATAACCCAGT-3′ and 5′-CCTATGACACGTATTTTGTGAAAGT-3′. The restriction endonuclease* Tth111*I (New England Biolabs, UK) was used to digest the PCR products at 65°C for 4 hours.

### 2.10. Statistical Analysis

Q-PCR data are reported as the mean ± SEM. Statistical analysis was performed by Student's* t*-test and by ANOVA. *P* < 0.05 was regarded as statistically significant. We also tested the relationships between continuous predictors (women's age, stress) and infant weight, using Pearson correlations and partial correlations (when adjusting for age and BMI) and using * t*-tests for dichotomous predictors (e.g., planned pregnancy) and ANOVA for testing the relationship between attitude type and infant birth weight, while controlling statistically for relevant confounders such as mothers' age, BMI, pregnancy planning, and pregnancy nutritional habits.

## 3. Results

### 3.1. Expression of Placental GAS5 and GRs

**Figure 1:**
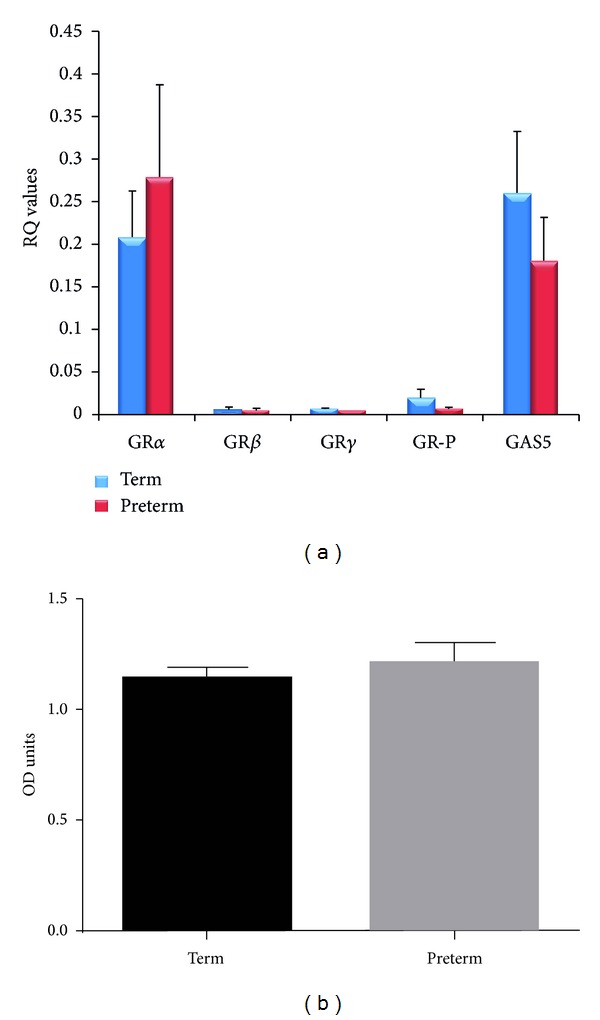
(a) Expression of GR*α*, GR*β*, GR*γ*, GR-P, and GAS5 in term (blue; *n* = 13) and preterm (red; *n* = 10) placentas. (b) Protein expression of GRs correcting over GAPDH, in term (*n* = 4) and preterm (*n* = 3) placentas.

Quantitative RT-PCR revealed that GAS5 and all GRs were expressed in human placentas (*n* = 23); 13 were preterm labor (<37 gestational weeks) and 10 were term (>37 gestational weeks) labor, with GAS5 and GR*α* being the predominant transcripts. In term labor, GR*α*'s ΔCt was 0.244 compared with 0.006 for GR*β*, 0.006 for GR*γ*, 0.023 for GR-P, and 0.26 for GAS5. At preterm, the values were 0.278, 0.004, 0.003, 0.006, and 0.180 for GR*α*, GR*β*, GR*γ*, GR-P, and GAS5, respectively (see Figure 1(a)). It is evident that the major transcripts in the human placenta are GR*α* and GAS5. When relative mRNA abundance was calculated, an approximate 4-fold change in the GR*α*/GR-P in the preterm labor group was noted compared with the term group. In addition, a twofold increase in the GR*α*/GAS5 ratio was also noted in the preterm group compared to the term group (see Table 2).

In term placentas, there was a significant correlation between GR*β* and GR*γ* (*r* = 0.580, *P* = 0.038) and with GR-P (*r* = 0.980, *P* < 0.001). In the preterm group, the dynamics of GR splicing were altered, since GR*α* correlated with GR-P (*r* = 0.710, *P* = 0.021) and GR*β* correlated with GAS5 (*r* = 0.792, *P* = 0.011). However, no significant correlation was noted in the preterm group between fetal weight or maternal stress and the relative expression of GRs and GAS5. In the term group, the only significant correlation was between GAS5 and maternal stress (*r* = −0.711, *P* = 0.021).

Due to ethical restrictions, we were only able to assess the expression of GR*α*/*β* in 4 term and 3 preterm placentas. Since GR is present as different isoforms, multiple bands were observed and there was an expected interpatient variation in the protein expression. Scanning densitometry of the bands corresponding to GR*α*/*β* normalized over GAPDH revealed no apparent differences in the expression of these variants between the term and the preterm groups (Figure 1(b)).

### 3.2. Cellular Distribution of GR*α* and GAS5 in Human Placentas

**Figure 2:**
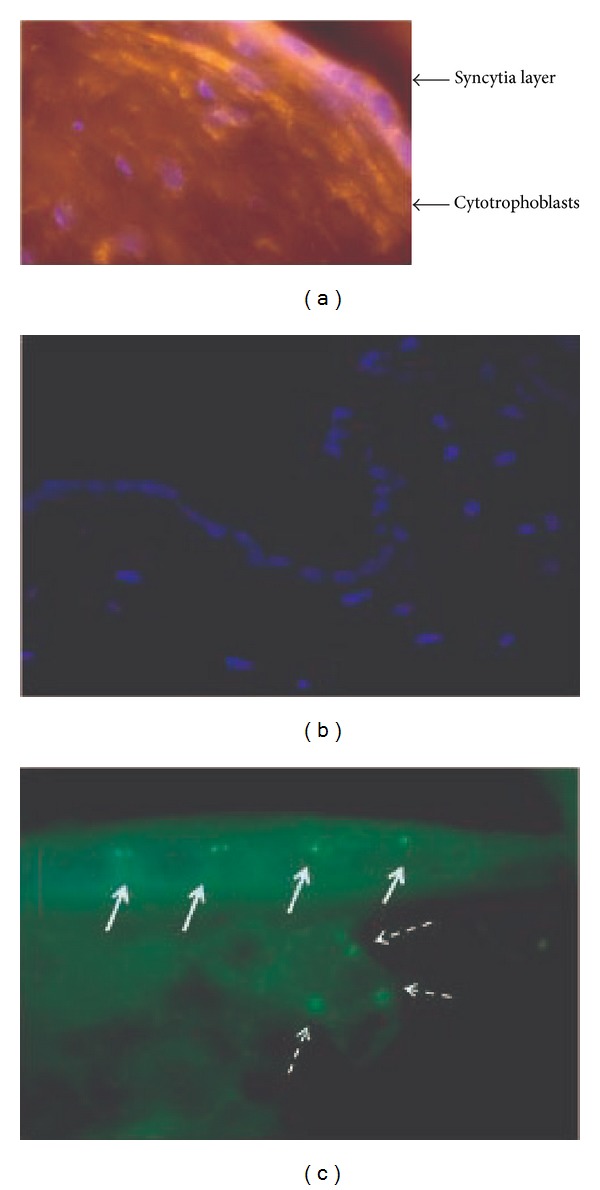
(a) Immunofluorescent analysis demonstrated expression of GR*α* primarily in the syncytiotrophoblastic layer of term placentas. (b) Negative control, confirmed specificity of immunostaining. (c) RNA FISH confirmed expression of GAS5 in cytotrophoblasts cells (dotted arrows) and syncytiotrophoblasts cells (white arrows).

Immunofluorescence analysis of the GR*α* protein was performed in human placenta tissue sections. Strong homogeneous staining mainly in the cytoplasm is detected in the syncytiotrophoblast cells on the outermost layer of the placental villi, with some scattered expression in cytotrophoblast cells (Figure 2(a)). Using RNA FISH, GAS5 localized primarily in syncytiotrophoblasts (Figure 2(c)). This is a first time that GAS5 localization has been studied in human placentas.

### 3.3. Effects of Cortisol on GRs and GAS5* In Vitro*

**Figure 3:**
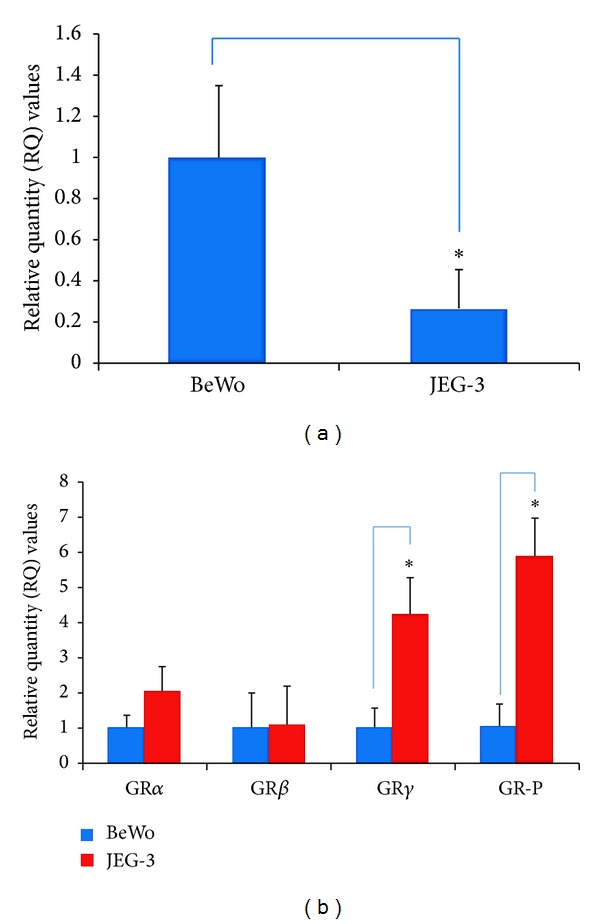
(a) Expression of GAS5 in BeWo and JEG-3 cells; *P < 0.05. (b) Expression of GR*α*, GR*β*, GR*γ*, and GR-P in BeWo and JEG-3 cells; *P < 0.05.

BeWo and JEG-3 cells were treated overnight with cortisol, to mimic moderate and high stress environments* in vitro*. Before doing so, the expression of GAS5 and GRs was examined in BeWo and JEG-3 cells without administering cortisol. GAS5 was significantly lower in expression in JEG-3 cells when compared to BeWo under basal conditions (Figure 3(a)). For the GRs, significantly higher expression levels were detected only for GR*γ* and GR-P in the JEG-3 cells compared to the BeWo cells (Figure 3(b)).

**Figure 4:**
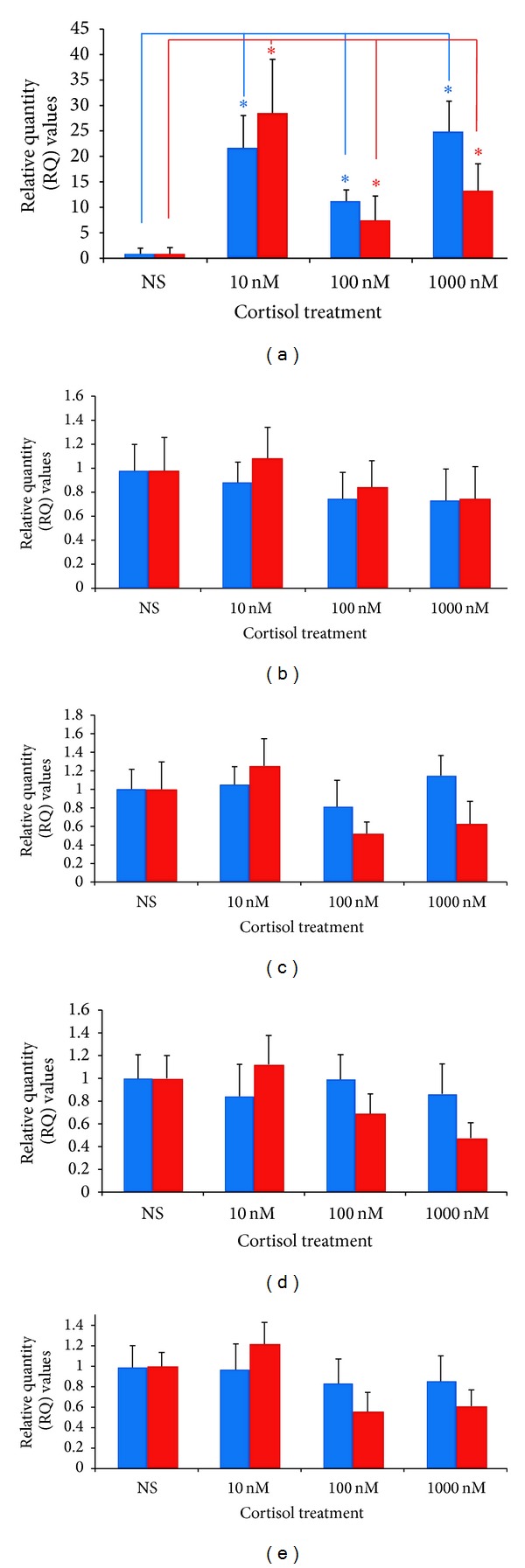
Effects of cortisol on the expression of GAS5 (a), GR*α* (b), GR*β* (c), GR*γ* (d), and GR-P (e) in JEG-3 (red) and BeWo (blue) placental cell lines. NS: no supplement, *P < 0.05.

When BeWo and JEG-3 cells were treated with cortisol 10 nM, 100 nM, or 1000 nM, the expression of GAS5 was significantly upregulated (all *P* < 0.05) compared with their corresponding basal levels (Figure 4(a)). The maximal stimulation for GAS5 was at 10 nM of cortisol in JEG-3 cells and at 1000 nM in BeWo. None of the cell lines exhibited dose dependency. However, cortisol treatments did not exert any significant changes in the gene expression of GR*α* (Figure 4(b)), GR*β* (Figure 4(c)), GR*γ* (Figure 4(d)), and GR-P in either of the cell lines (Figure 4(e)). In addition, there were no apparent changes in protein expression of GR*α*/*β* in BeWo cells treated with 10–1000 nM of cortisol, corroborating the qPCR studies (data not shown).

### 3.4. Maternal and Infant GR Polymorphisms, Maternal Attitudes, and Fetal Birth Weight

Four GR polymorphisms were investigated:* Bcl*I, N363S,* Tth111*I, and ER22/23EK (Table 3). Since the maternal attitude towards the pregnancy was a significant predictor of fetal birth weight, we reexamined this association as a function of the GR genepolymorphisms, resembling a gene-environment interaction and statistically controlling for mothers' age, BMI, pregnancy planning, and pregnancy nutritional habits, to remove any potential sources of statistical bias that could skew our data.

Statistical analysis of the maternal* Tht111*I polymorphism has shown an inverse correlation between negative maternal attitude and infant birth weight (*r* = −0.41, *P* = 0.030), only in* Tht111*I CC polymorphism subgroup. In* Tht111*I CT, no significant correlations were noted, controlling for age, BMI, pregnancy planning, and consumption of fast food during pregnancy. These data point towards an interaction between stress and genetics, since only in the CC polymorphic GR group did negative maternal attitude predict fetal weight reduction, but not in the CT group, independent of confounders. Hence, the effects of maternal attitudes on fetal weight depend on the mother's polymorphism of GR gene. None of the remaining GR polymorphism subgroups demonstrated any differential correlations between maternal attitudes and fetal weight (data not shown).

## 4. Discussion

The present study extends previous findings and provides evidence for the first time how maternal stress and GR polymorphisms can potentially affect fetal outcome together. As reviewed previously, prenatal maternal stress has been shown to have long-term effects on the psychological as well as behavioral development of the offspring [15, 16]. In our cohort, women with negative attitudes exhibited higher levels of stress during pregnancy compared to women with neutral/positive attitudes and gave birth to infants with lower birth weights than those with positive/neutral attitudes towards their pregnancy (500 gr difference [13]).

In terms of the polymorphisms analyses, only the maternal* Tth111*I polymorphism was suggestive of a gene-environment interaction since, only in* Tth111*I (CC), negative versus positive/neutral maternal attitudes towards the pregnancy predicted fetal weight reduction, but not in the* Tth111*I (GC) group, independent of important confounders. These confounders included women's age and BMI and were not explained by gestational age. Additionally, multiple other immune symptoms and demographic variables tested were also not predictive of fetal weight. This is the first time that a gene-environment interaction between a GR polymorphism and maternal attitudes towards pregnancy was found, in relation to fetal weight. To this date there has been some contradicting evidence as to the role of these polymorphisms in fetal outcomes [17–19].

The human placenta is exposed to increased levels of cortisol as pregnancy progresses [20] and its effects are mediated via binding and activating the GR. However, controversy surrounds the exact mechanisms by which these responses are regulated. GR alternative splicing might also influence the subsequent activation of signalling pathways by glucocorticoids [20]. In our study, we have shown that all known transcripts of GR splice variants are expressed in the human placenta with GR*α* being the predominant transcript in all categories studied. These data corroborate a previous preliminary study of placental GRs [20]. Moreover, we demonstrate for the first time the expression of GAS5 in human placentas. We decided to incorporate GAS5 in the current study as it can act as a GR DNA binding decoy and as a result compromise its activity [8]. Interestingly, GAS5 and GR*α* were the predominant transcripts in both term and preterm placentas.

Here, we provide further information about the regulation of this transcript by cortisol using two* in vitro* models as it is upregulated by cortisol in a dose-independent manner. This finding provides further evidence of regulation of GAS5 by stress and corroborates initial* in vivo* data in mice [21]. In this study using C57BL/6 male mice, stress induced GAS5 RNA levels in the hippocampus and this increase was accompanied by a rise of corticosterone levels [22]. These* in vitro* and* in vivo* observations together are highly suggestive of a functional link between stress and this ncRNA. Moreover, when we performed RNA FISH, the GAS5 transcript was localized almost exclusively in the syncytiotrophoblast layer of the human placenta and colocalizes GR*α*. In view of previous data in HeLa cells, where GAS5 translocates from the cytoplasm into the nucleus with GR in response to dexamethasone [8], this colocalization is highly suggestive of a potential crosstalk at placental level between GAS5 and the GR.

Preterm labor is associated with high mortality and morbidity [22] and a recent study pointed towards an association between maternal stress and complications of pregnancy, especially preterm birth [23]. GR*β* differs from GR*α* on the C-terminus of the receptor protein. There is some controversy surrounding the exact function of GR*β*, but it appears to exert a dominant-negative effect on GR*α*-induced transcriptional activity [8]. Moreover, it fails to bind glucocorticoids and activate subsequent transcriptional events [24]. GR*γ* is a ligand-dependent transcription factor with reduced transactivating activity and its function is still under investigation. In our clinical samples, GR*γ* is present at much lower levels than the GR*α* isoform in the placentas studied. Similarly, little is known about the role of GR-P, a truncated isoform that lacks a large part of the ligand-binding domain, including the domains for silencing of GR in the absence of hormone and transcriptional activation [24].

In our study, an approximate 4-fold change in the GR*α*/GR-P in preterm labor was detected. Johnson et al. have shown that placental GR-P mRNA levels were reduced significantly after spontaneous labor [20]. A twofold increase in the GR*α*/GAS5 ratio was also noted in the preterm group compared with the term group of women. It is attractive therefore to hypothesise that the change in the ratio of the splicing isoforms alters the responsiveness of placental GRs to cortisol and that this may affect gestational age. We would like to propose a potential model where, during preterm birth, GR*α* is the predominant receptor since there is a decrease in negative regulators such as GR*β*, GR*γ*, and the “pseudo-GRE” GAS5. These ratio changes will ultimately lead to an augmented response towards glucocorticoids with potential detrimental effects for the mother and the fetus. It is possible that this change of relative transcript abundance ratio might be involved in the correlation between negative maternal attitudes (hence highly stressed) and fetal birth weight.

In terms of the polymorphic study, our study provides a novel insight into the involvement of GR polymorphisms in pregnancy outcome. We have identified a specific group of mothers (Tht*III*I polymorphism, CC group) in whom maternal attitudes predicted fetal weight. In a past study, a similar gene-environment interaction was noted between the ER22/23EK polymorphism and the effect of childhood adversity on depression [17].

We acknowledge that measurement of GR polymorphisms posed certain limitations, for example, the use of cell lines as* in vitro* experimental models. Although both cell lines (JEG-3 and BeWo) could represent a cytotrophoblastic milieu* in vitro*, they have differences in their fusogenic capacities. In addition, microarray analyses demonstrated that many transcripts were differentially expressed between JEG-3 and BeWo cells [25]. It would be of interest to repeat these experiments in primary cell lines of syncytialised trophoblasts. This experiment would provide a better insight into the regulation of GAS5 by cortisol. However and despite these limitations, there is a wealth of literature using those two cell lines as experimental models to study placental function. It would also be of interest to expand these observations in human myometrium that is a key organ responsible for quiescence and contractility responses and also assess whether changes in GR transcripts are due to labor or nonlabor. To test this, we have performed qPCR for all GRs and GAS5 in the same cohort of placentas divided this time to labor (*n* = 18) and nonlabor (*n* = 5). There was no apparent change in the expression of any of the genes in these two categories (data not shown). Therefore, at placental level, the contractile status does not really affect GR transcription.

We acknowledge that our sample included small numbers in certain categories of polymorphic groups. Moreover, the nature of maternal stress was not identified and further analytical approaches are needed to provide conclusive evidence for a gene-environment interaction. Nevertheless, the statistically significant effect and the size of differences observed between mothers with negative versus positive and neutral attitudes in the* Tht111*I CC polymorphism group suggest that this effect may be robust. Second, the distribution of* Bcl*I polymorphism seen in this sample may be unique, since there are important geographical/ethnic differences in the prevalence of these polymorphisms. It should be emphasized that, in the present study, we included a fairly homogeneous cohort of Mediterranean patients from Crete. Despite these limitations, this is the first study to demonstrate a gene-maternal environment synergism in relation to infant birth weight, using a very brief assessment of maternal attitudes to pregnancy. Should these data be replicated in a much wider cohort, given the simplicity in assessing such attitudes and the feasibility to identify the homozygous group of women early on in pregnancy, these findings may have significant implications for public health and prevention. For example, based on the polymorphic profile and our brief assessment of mothers' attitude towards the pregnancy, we could screen noninvasively and identify mothers during pregnancy that may benefit from stress-management strategies, and this could possibly dramatically improve health outcomes for the mother as well as the fetus.

## Figures and Tables

**Table 1:** Sequences of primers used for quantitative RT-PCR.

GR*α*	Sense	5′-CTATGCATGAAGTGGTTGAAAA-3′	96 bp
	Antisense	5′-TTTCAGCTAACATCTCGGG-3′	

GR*β*	Sense	5′-GAAGGAAACTCCAGCCAGAA-3′	81 bp
	Antisense	5′-CCACATAACATTTTCATGCATAGA-3′	

GR*γ*	Sense	5′-TTCAAAAGAGCAGTGGAAGGTA-3′	264 bp
	Antisense	5′-GGTAGGGGTGAGTTGTGGTAACG-3′	

GR-P	Sense	5′-GCTGTGTTTTGCTCCTGATCTGA-3′	194 bp
	Antisense	5′-TGACATAAGGTGAAAAGGTGTTCTACC-3′	

GAS5	Sense	5′-CAGTGTGGCTCTGGATAGCA-3′	168 bp
	Antisense	5′-TTAAGCTGGTCCAGGCAAGT-3′	

*β*-Actin	Sense	5′-AAGAGAGGCATCCTCACCCT-3′	216 bp
	Antisense	5′-TACATGGCTGGGGTGTTGAA-3′	

**Table 2:** Relative mRNA abundance of GR*α* over GR*β*, GR*γ*, GR-P, and GAS5.

Ratio	Term (*n* = 13)	Preterm (*n* = 10)
GR*α*/GR*α*	1	1
GR*α*/GR*β*	47	65
GR*α*/GR*γ*	38	97
GR*α*/GR-P	11	43
GR*α*/GAS5	0.8	1.5

**Table 3:** Distribution of GR polymorphisms.

Polymorphism	Nucleotide change	Genotype	Distribution (%)
			*n* = 81
*Bcl*I	C → G	GG	5 (6.2)
		CC	19 (23.5)
		GC	57 (70.4)

			*n* = 81
*N363S *	Asp → Lys	wt	79 (97.5)
		Hmz	2 (2.5)

			*n* = 81
*ThtIII*I	C → T	CC	35 (43.2)
		TT	2 (2.5)
		CT	44 (54.3)

			*n* = 81
*ER22*/*23EK *	Arg → Lys	wt	78 (96.3)
		Htz	3 (3.7)
